# Integrating a FISH imaging system into the cytology laboratory

**DOI:** 10.4103/1742-6413.62258

**Published:** 2010-04-06

**Authors:** G. Denice Smith, Matt Riding, Kim Oswald, Joel S. Bentz

**Affiliations:** 1 ARUP Institute for Clinical and Experimental Pathology^®^, Associated Regional and University Pathologists (ARUP) Laboratories, Inc., Salt Lake City, Utah, USA; 2 Department of Cytology, Associated Regional and University Pathologists (ARUP) Laboratories, Inc., Salt Lake City, Utah, USA; 3 Technical Operations, Associated Regional and University Pathologists (ARUP) Laboratories, Inc., Salt Lake City, Utah, USA; 4 Department of Pathology, Associated Regional and University Pathologists (ARUP) Laboratories, Inc., Salt Lake City, Utah, USA; 5 Laboratory Medicine Consultants, Ltd., Las Vegas, NV, USA

**Keywords:** Automated screening, microscopy, bioview duet, cytotechnologists, fluorescence *in situ* hybridization, image-analysis, image-processing, UroVysion FISH

## Abstract

We have implemented an interactive imaging system for the interpretation of UroVysion fluorescence in situ hybridization (FISH) to improve throughput, productivity, quality control and diagnostic accuracy. We describe the Duet imaging system, our experiences with implementation, and outline the financial investment, space requirements, information technology needs, validation, and training of cytotechnologists needed to integrate such a system into a cytology laboratory. Before purchasing the imaging system, we evaluated and validated the instrument at our facility. Implementation required slide preparation changes, IT modifications, development of training programs, and revision of job descriptions for cytotechnologists. A darkened room was built to house the automated scanning station and microscope, as well as two imaging stations. IT changes included generation of storage for archival images on the LAN, addition of external hard drives for back-up, and changes to cable connections for communication between remote locations. Training programs for cytotechnologists, and pathologists/fellows/residents were developed, and cytotechnologists were integrated into multiple steps of the process. The imaging system has resulted in increased productivity for pathologists, concomitant with an expanded role of cytotechnologists in multiple critical steps, including FISH, scan setup, reclassification, and initial interpretation.

## INTRODUCTION

The UroVysion Bladder Cancer Kit detects common chromosomal aberrations associated with bladder cancer by fluorescence *in situ* hybridization (FISH).[1] Initially approved in 2001 as a monitor for bladder cancer recurrence, the UroVysion Bladder Cancer Kit was approved by the FDA in January 2005 for initial diagnosis of bladder cancer. Our laboratory has offered the test since 2002. By January of 2007, the UroVysion FISH volume was increasing steadily, with each case requiring 30 minutes of pathologist time for sign-out. In addition to cost-effectiveness concerns, we wanted to improve FISH quality control by having trained cytotechnologists determine hybridization quality prior to the slides reaching the pathologists desk. We felt an imaging system might provide a solution and the tools to address these and additional issues that are outlined in Table 1.

**Table 1:** Goals for integrating imaging system for FISH in cytology laboratory

*Issue*	*Description of issue*	*How imaging system might help*
Accuracy	Contributing to the challenges of manual FISH interpretation are slide variations in probe signal strength and background fluorescence	Under the low light level conditions typical for FISH, advanced digital imaging detectors and image processors offer advantages over the human eye, including greater sensitivity and resolution. The goal would be to reduce occurrences of false positives and false negatives
Pathologist time	Manual screening required up to 30 min/case	Interactive automated screening would provide for optimized images of cells classified into normal and abnormal categories for pathologist review
Expanded roles for cytotechnologists	As cytotechnology evolves to incorporate molecular technologies, it is desirable to integrate and benefit from the skills and knowledge of cytotechnologists	Interactive automated screening would involve cytotechnologists, who would prepare the case, with troublesome cells highlighted for a pathologist to review
Quality control	Issues with hybridization quality result in turn-aroundtime delays if not detected soon after completion of FISH	Involvement of qualified morphologists (cytotechnologists) would allow for quality control at the completion of FISH
Interactive live microscopic review of relocated cells of concern	Some troublesome cells with apparent polysomy could represent overlapping cells. Live microscopic review of these cells would be appropriate, particularly for borderline cases	Interactive imaging systems record coordinates of cells for relocation and live examination under a microscope
Image archiving	There are CAP LAP requirements for image archiving that must be satisfied for CLIA laboratory accreditation	An interactive imaging system would provide a mechanism for image archiving
Location-guided screening	Sequential localization of abnormal cells by urine cytology (morphology), followed by UroVysion FISH, would provide adjunctive information for equivocal or indicative cases	An interactive imaging system would provide a mechanism to revisit cells of concern following FISH
Research applications	Sequential localization of cells by cytology or ICC, followed by FISH; data acquisition and storage; permanent record of data, etc.	Target FISH (ICC followed by FISH, etc.) is supported by the Duet imaging system, with software for data acquisition, etc.

A number of imaging systems are available for FISH and other applications relevant to cytology, but the Duet[2] and the Ikonisys systems[3] are the only systems currently that have been FDA cleared for automation of UroVysion FISH for bladder cancer interpretation. We implemented the Duet imaging system (BioView, Ltd.), with the goals of improving throughput, productivity, quality control and accuracy, as well as reducing the evaluation time required of pathologists by having cytotechnologists perform the initial examination.

This overview provides information that may be valuable for other labs considering implementation of automated-imaging to aid in UroVysion FISH interpretation. Although focusing on one particular imaging system, many of the issues will be shared in common with other systems. We include an overview of Duet and the general workflow steps involved in UroVysion FISH and image-aided interpretation. We also summarize costs, space, IT, validation, and training issues that were involved in implementation. The introduction of new tests or instrumentation into a lab requires careful consideration of how a test will benefit patients, cost-effectiveness, and the needs and capabilities of the laboratory. An extensive literature in this area can provide suggestions and checklists to guide labs through the process.[4–7]

## OVERVIEW OF UROVYSION FISH AND DUET IMAGING

The UroVysion Bladder Cancer Kit is FDA-approved as an aid for initial diagnosis, or to monitor for recurrence, of bladder cancer. The multi-color, 4 -probe mixture can detect amplifications of chromosomes 3, 7, and 17, or the deletion of 9p21, the locus of the tumor suppressor gene, P16^INK4a^.[8–14] UroVysion FISH probe signals are scored during interpretation, requiring the ability to recognize abnormal urothelial cells, whether evaluation is performed manually or with the aid of an imaging system.

For UroVysion FISH target cell classification, the Duet imaging software defines nuclei using DAPI stain, which is also used to determine the order of target cells scanned, with large, elliptical, patchy nuclei visited first. The BioView Duet imaging system scans the slide, and finds, classifies, and captures images of cellular targets, which are then presented in a gallery on a screen for review.

The imaging system consists of a Duet scanning station as well as one or more Solo reviewing stations. The Duet station controls an automated microscope, with a stage that can be positioned in X, Y, and Z (up and down) axes with micrometer-level precision. This allows the software to control the stage to focus on cells, and retain coordinate information for the specific locations of target cells. A Z stacking feature controls the number of levels that are captured as the stage is moved up-and-down through different focal planes. The system controls automated objective and filter turrets, as well as a frame grabber and a 3-CCD (charge coupled device) full-color camera, with a sensor chip necessary to convert light to electrical current and ultimately to a digital image.

### Workflow steps

**Figure 1:**
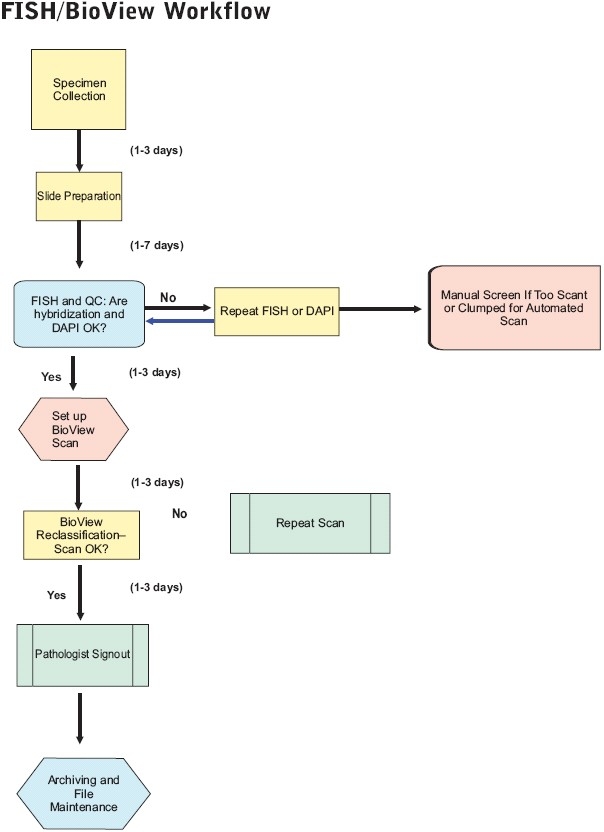
Workflow steps. Steps involved in processing and completing UroVysion FISH cases are shown, including specimen collection, slide preparation, FISH, scan setup, reclassification, pathologist interpretation, and archiving and file maintenance

Workflow steps for UroVysion FISH and Duet-aided interpretation are summarized in Figure 1. Cytotechnologists perform many of the steps, including slide preparation, FISH, scan setup, and reclassification. Steps involving FISH and the imaging system are summarized in more detail below. Further details on all aspects, including specimen collection, transport, and slide preparation, can be provided on request.

#### Slide preparation

**Figure 2:**
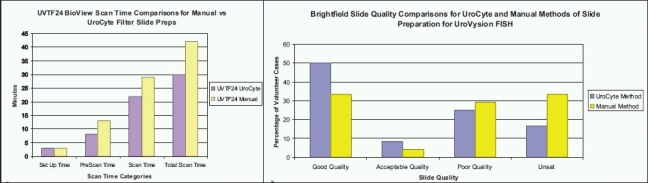
Scan time and slide quality comparisons. a) Scan time comparisons for manual vs UroCyte filter slide preps for one split urinary sample. b) Brightfield slide quality comparisons for UroCyte and manual slide preparation methods for UroVysion FISH

We recently validated the use of ThinPrep UroCyte filters for samples in PreservCyt, a slide preparation method previously described.[15] The UroCyte-prepared slides show less clumping, as well as improved cellularity, morphology, and scan times compared to the manual method [Figure 2], which is described in the UroVysion Bladder Cancer Kit package insert available on request from Abbott Molecular.

#### UroVysion FISH

UroVysion FISH hybridization steps were previously carried out exclusively by medical technologists in another department. With the switch to Duet-aided screening and an increased volume in UroVysion FISH, cytotechnologists were cross-trained to carry out FISH hybridization and wash steps in a second run during the week. This has improved turnaround time (TAT), and increased opportunities for quality control and troubleshooting. Because the cytotechnologists have morphological expertise with training to screen UroVysion FISH, problems with weak hybridization on control and patient slides can be quickly recognized and sent back for re-probing.

#### Loading and scanning

**Figure 3:**
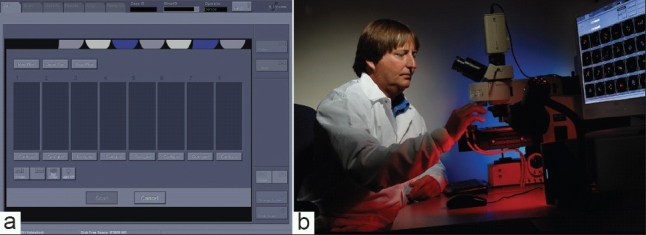
Loading slides onto the Imaging System. a) Duet computer interface for loading slides for an automated scan. b) Cytotechnologist loading UroVysion FISH slides onto modified stage for automated scan

To set up a scan, slides are loaded into the eight slots on the modified stage, including positive/negative control slides. Loading is important because the ability of the system to accurately relocate target cells depends on the precise placement of the slide, and although not difficult, does take some practice. The scan is set up using the system computer interface [Figure 3a], which displays an image of the eight slot slide holder. Slide/patient information is entered and scan parameters are set independently for each slide in a run. Because it is important to identify urothelial cells and the quality of hybridization for quality control, cytotechnologists have been trained to set up the imaging scans [Figure 3b].

**Figure 4:**
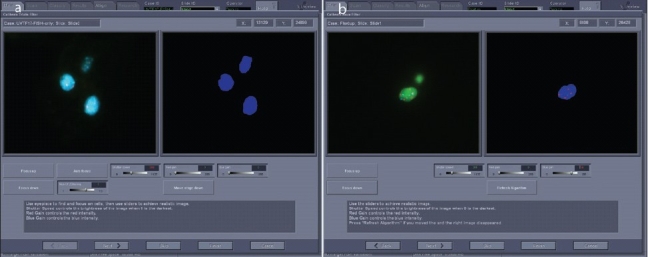
Adjusting gain and shutter speed for optimized scan setup. a) The DAPI signal is optimized for the system to detect cell nuclei. The image on the left is similar to what would be seen through the oculars. On the right is a representation of what the system recognizes as nuclei. b) Gain and shutter speeds are adjusted for CEP3 SpectrumRed and CEP 17 SpectrumAqua signals. On the left is an image similar to what would be seen through the microscope; on the right is a summary view of what the system “sees”.

**Figure 5:**
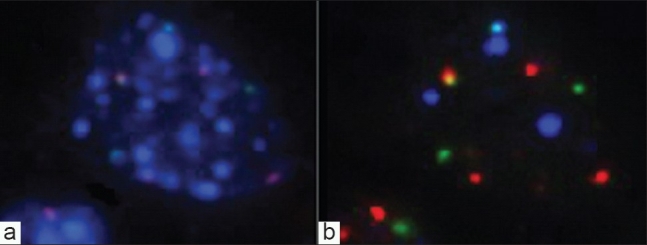
DAPI and probe signal patterns for an abnormal cell. a) Abnormal cell with typical patchy pattern of DAPI fluorescence. b) Combined probe patterns showing abnormal numbers of red, green, and blue signals.

The cytotechnologist looks over the field of cells on a patient slide and selects a urothelial cell (abnormal if present) to adjust the system settings. Gain and shutter speed camera adjustments are made for each filter sequentially, beginning with the DAPI filter [Figure 4a] followed by the other filters [Figure 4b]. Because the DAPI image is used by the system to define and identify what is a cell, it is important that the settings be adjusted so that abnormal urothelial cells, which may have weak or patchy fluorescence, will be detected [Figure 5]. After adjustments have been made for all slides to be included, a click on the Scan icon initiates a scan.

The entire setup process should take 2–3 minutes per slide, but training and practice are required to set up reliable, good quality scans. A software update is available to increase the speed of scans, as well as automatically adjust the settings for a scan.

#### Cytotechnologist reclassification

For UroVysion FISH, a positive interpretation requires four or more cells displaying an excess of more than two signals for two or more of the probes for chromosomes 3, 7, or 17, or a homozygous deletion for 9p21 in 12 or more cells. In addition to the absence of abnormal cells, a negative BioView interpretation requires that 100 cells be classified as normal.

**Figure 6:**
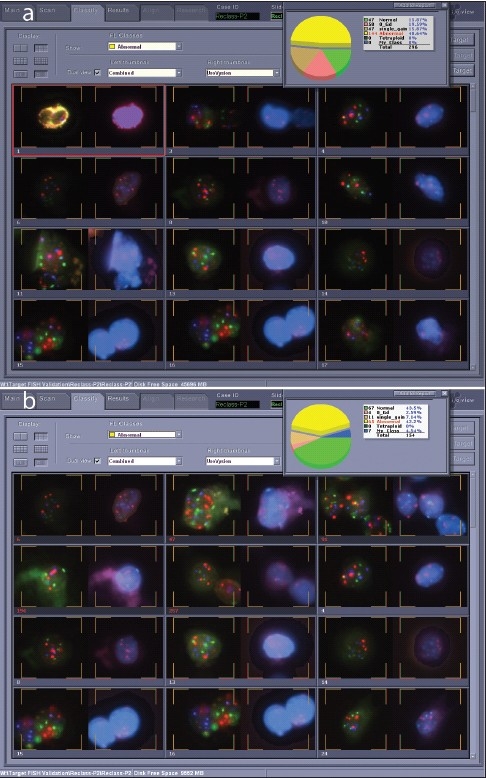
Target cell gallery before and after reclassification. a) Target cell gallery before reclassification, showing 144 target cells in the abnormal category and only 47 normal cells for this case. b) Target cell gallery after reclassification. Seventy percent of the target cells for this case were shifted into other categories during reclassification. This case was clearly positive, and full reclassification would not be necessary once four abnormal cells were identified.

**Figure 7:**
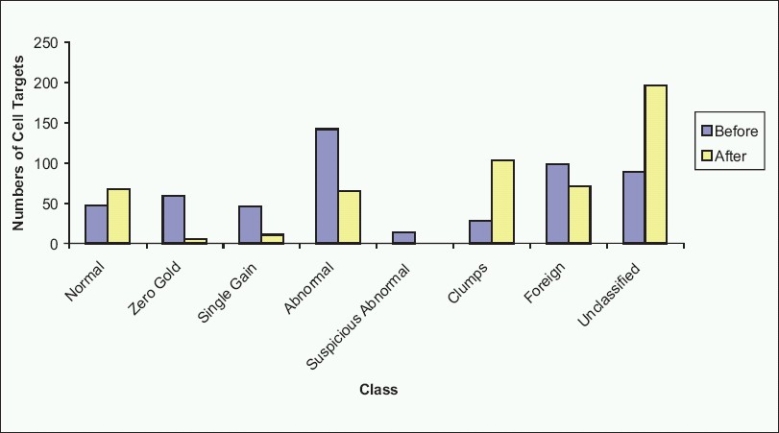
Cell targets before and after reclassification. As can be seen in this example, reclassification by a cytotechnologist results in changes to the numbers of target cells in each class, organizing the case for interpretation

The software performs image-analysis on captured target cells, and attempts to classify the cells into various categories based on nuclear size and shape, as well as probe signal enumeration. Many variables are involved in making this determination. In our lab, a trained cytotechnologist checks the classification at the completion of a scan, and reclassifies as needed using a Solo work station. Figure 6 displays an example, with images of the software interface from a case before [Figure 6a] and after [Figure 6b] reclassification. The pie chart shows that 296 target cells were initially sorted by the software into normal (two signals for each of the four probes present), zero gold (two signals for CEP3, CEP7, and CEP17, but no signals for LSI 9p21), single gain (2 signals each for three for the probes, but more than two signals for one probe), and abnormal (>2 signals for two of the CEP3, CEP7, or CEP17 probes) classes, with about half of the targets in the abnormal class, and the remainder split roughly equally into the other three classes. At the completion of reclassification, normal and abnormal classes were roughly equal large slices of the pie [Figure 6b]. In this instance, 70% of the original classified target cells required reclassification [Figure 7].

The cytotechnologist thus reorganizes scan data into a form that is readily interpretable by the pathologist, with normal and abnormal cells clearly segregated for review. After offering a preliminary interpretation and recording the case in a log stored by the work station, the cytotechnologist notifies the pathologist that the case is ready for review.

#### Pathologist sign-out

Pathologists can sign-out UroVysion FISH cases on-site using a Solo station or using Solo software installed on their computers. A pathologist examines the reclassified cases, and gives an interpretation of positive or negative where possible. Although cases are reclassified by a cytotechnologist to allow the pathologist to focus on abnormal cells, all of the target cells from the original scan are still available for the pathologist to review, but in an organized format that makes it easy to scan through the classes rapidly.

Although a pathologist often signs-out a case using only the Duet-generated images, the imaging system provides the tools, if desired, to examine the actual slide through a microscope. If there are cells-of-concern that could represent possible polysomy or, alternatively, two overlapping nuclei, it is possible to review them under the microscope using the software in Interactive mode to relocate the cells. In those cases for which a definitive interpretation of the slide cannot be made after pathologist review, three options are recommended for resolving the case, including direct examination of questionable target cells, rescanning the case to include more target cells, or a manual rescreening of the slide.

#### Post sign-out activities: image archiving, hard drive maintenance, slide and record retention

##### Slide storage and image archiving

UroVysion FISH slides in our lab are retained at −20°C for a period of more than three months from the time of interpretation. Although currently the College of American Pathologists (CAP) Laboratory Accreditation Program requires archiving two images on positive cases and one image on negative cases, we archive four images on positive cases and two images on negative cases. The archived images, which use only a few megabytes of memory, are exported to the LAN where they are backed up and stored for 20 years, a CAP requirement (CYG.32700). Ultimately we hope to export the images directly to diagnostic reports.

##### Managing case files, images, and hard drives

The entire set of data and images that make up each case may require 0.5-1 GB of storage space. The system automatically saves each case to two parallel hard drives. Because scan times are impacted by storage capacity, it is not possible to retain signed-out cases for long periods of time on the instrument hard drives, which each have a case storage capacity of 212 GB. After archiving select images, cases are deleted from the system.

One of our goals is to develop a storage system to permit long-term archiving of a library of scanned cases for educational/training and research purposes. Some select cases have been copied before reclassification for training purposes, but many informative and interesting cases have been deleted out of necessity. We recommend setting up a system to save cases to two storage hard drives – one in which the case is preserved in an unmodified, original state right after completion of a scan; the other after reclassification and sign-out.

Maintaining the system hard drives is a never-ending task. Research and validation cases, as well as clinical cases for which images have not yet been archived, are currently backed up using an external hard drive and DVDs. The time needed to copy and delete files is considerable, and requires scheduling this task for a time when the instrument is not in use for scans.

## SPECIAL CONSIDERATIONS FOR IMPLEMENTATION OF AN IMAGING SYSTEM INTO THE LAB

A number of important issues to consider before bringing an imaging system into a lab include the expense of the system, space and IT requirements, the need to perform an adequate validation, and the time and resources required for adequate training and continuing education.[4–7] It was one full year from the time the instrument arrived for evaluation until it was implemented for clinical cases. The extensive validation study we performed provided time to work out IT and space issues, as well as ample pathologist experience with the system. Cytotechnologists began training six months before implementation, but in the context of a normal workload with periods of time when there was no training. If implementation had occurred sooner, the process could have been modified to speed up the training.

### Expense and space requirements

Some of the areas in which there were cost outlays or savings associated with the implementation of the imaging system are summarized in Table 2. Although our specific costs are not given because of institutional policies, the list does provide indications of costs to be expected, assuming that the UroVysion FISH test with a manual screen is already offered by a lab. One of the unanticipated costs was the need to build a darkened room to house the Duet and two Solo stations. A thorough cost/benefit analysis has not been done subsequent to implementation.

**Table 2:** Possible costs and benefits of implementation of duet to aid in the interpretation of UroVysion FISH

*Cost/Benefit area*	*Description*
Imaging system: Hardware and software	
Duet	Scanning station with microscope, computer, monitor, and associated peripherals.
Solo stations and keys	Two Solo stations for reclassification, as well as Solo software for pathologists' computers, were purchased.
System upgrades	Software and hardware upgrades are available
Construction of room to house duet and solo stations	The BioView requires that scans be done in a darkened room. We built a room approximately 12' × 6' which houses the BioView Duet and two Solo stations. Although the Solo stations can be placed anywhere, the cytotechnologists prefer to reclassify in a darkened room
Switch to UroCyte filter prepared slides	
Reagents and supplies	Increased costs associated with UroCyte method, including filter, reagents, and supplies
Cytology lab labor costs	Decreased personnel costs associated with shorter time required to prepare slides by UroCyte method
Cytotechnologist labor costs	Decreased personnel costs with UroCyte method because cytotechnologist does not need to monitor slide preparation
Duet vs. manual screening	
Cytotechnologist involvement	Added involvement of cytotechnologist in Duet-aided screening (scan setup, reclassification, and preliminary interpretation). This is offset by decrease in pathologist time.
Pathologist	Decreased personnel costs and increased productivity because of reduced time required to sign out case by a pathologist with use of Duet-aided interpretation. We estimate that incorporation of cytotechnologists and the Duet imaging system decreases pathologist effort in signing out cases by about 25 minutes per case. There may be increased accuracy as well
Scan times	Decreased scan times for UroCyte filter prepared slides; decreased TAT; increased productivity
Validation	
Personnel	R & D scientist/cytotechnologist
Reagents and supplies	If not using accessioned clinical cases, reagents and supplies are needed for slide preparation and FISH
Data storage	Back up storage devices
IT	
Back-up and storage	External or internal hard drives; space on LAN for archived images
Wiring/connection changes	Wiring/connection changes had to be made for communication between Duet and remote Solo stations
Personnel time	IT personnel
Training	
Personnel and educational materials	
Proficiency tests	Annual Abbott molecular proficiency panel and biannual CAP proficiency testing

Currently, the Duet system with the automated scan setup upgrade is listed at $139,000. CPT codes and reimbursements vary by state, but most states currently use 88367 (4 probes, Duet-aided interpretation), and 88368 (4 probes, manual interpretation) CPT codes. Ten states currently use 88299 (unlisted cytogenetic study) for UroVysion FISH, with a negotiated reimbursement.

### Validation

To validate, we compared manual and BioView-aided interpretations for 135 consecutive UroVysion FISH cases that were received in the cytology laboratory. We included this large number to adequately evaluate the system, to gain proficiency, and to resolve any IT, training or other issues before final implementation. Manual and Duet-aided interpretations were compared with respect to accuracy, between-run precision, and time required. The two-armed, blinded validation study comparing manual and Duet-aided UroVysion FISH in our lab is currently pending publication.

### Training

Initial training on the system was provided by BioView technical personnel. We subsequently have trained cytotechnologists as well as pathologists/fellows/residents. Our FISH/Duet training program provides both a strong theoretical foundation and practical experiences.

Cytotechnologists: After introductions to FISH and the Duet instrument, each cytotechnologist reclassifies 100 copied, unreclassified cases and reviews them with a pathologist. Experience with a significant number of cases is required to gain confidence and speed. Prior to independent reclassification of clinical cases, each cytotechnologist has to successfully reclassify and interpret three unknown cases.

Pathologists/fellows/residents: Because pathologists occasionally need to sign out cases manually, UroVysion FISH training begins with manual screening training sessions at a multi-head microscope and review of unknown slides, followed by a 10-slide proficiency test. Training for image-aided interpretation includes review of FISH cases with an experienced pathologist, followed by evaluation of unknown scanned cases. Proficiency is acquired by reclassifying scanned cases, as well as interpreting cases already reclassified by a cytotechnologist. The trainee subsequently interprets clinical cases independently with review by a more experienced pathologist. Prior to independent sign-out, the trainee must pass a 10-case proficiency test of unknown imaged cases using both manual and image-aided interpretation.

The training for pathologists and cytotechnologists is not trivial, particularly in the context of normal workload responsibilities, and we recommend preparing training materials in advance. Because scheduled lectures and training sessions may not occur when new individuals need training, a long-term goal is to have lectures, FISH training modules, and quizzes available online using e-learning software. Cytotechnologists have found it very helpful to review interesting or difficult cases with pathologists; these sessions will be recorded for repeated viewing.

### Proficiency testing

Pathologists and cytotechnologists participate in the biannual CAP proficiency testing, as well as the Abbott Molecular proficiency panel on an annual basis.

### IT issues

Competent IT support is critical at all stages, and IT concerns remain among our greatest challenges, particularly the limited storage capacity of the instrument hard drives.

For reclassification, we soon needed a second Solo station to deal with the FISH volume and cytotechnologist schedules. Because pathologists' offices are in a different building from the location of the instrument, Solo software was purchased for use on their office computers, and networked to communicate with Duet. Getting the software to work properly with the necessary wiring/cable changes proved more challenging and time-consuming than expected.

Institutionally-managed computer software and programs must be compatible with the imaging system, as we discovered when the Duet software would not run after a computer anti-virus security program was installed.

For instrument software issues, BioView technical personnel can access the Duet system, and remotely make adjustments to improve scan and other instrument parameters.

## SUMMARY

### Pathologist sign-out time, turnaround time, and throughput

The addition of automated imaging has provided the image-processing and target-sorting tools to optimize the cell images on which an interpretation is based. Our estimate is that incorporation of cytotechnologists and the Duet imaging system substantially decreases the pathologist time necessary to finalize a report. This has made it possible to manage a 66% increase in volume subsequent to implementation of the Duet imaging system.

Although the introduction of automated scan and reclassification steps might be expected to increase the time required to sign-out a case, it was hoped that it would ultimately result in not only savings to pathologists' time, but also an improvement in TAT. That did not happen initially, in part because of the time required to gain proficiency and confidence in the system, and in part because only one hybridization was being run a week which made it difficult to coordinate scanning, reclassification, and sign-out activities.

The current turnaround time ranges from six to 12 days, from collection to sign-out, but is expected to improve with system upgrades and an additional FISH run during the week. Currently we scan 16 UroVysion FISH cases over a 24 hr period, with eight cases run during the morning, and eight cases run overnight. We make the instrument available in the daytime for image archiving, research, and other activities, which limits throughput by restricting scans mainly to morning and overnight hrs. An available upgrade makes it possible to load up to 50 slides at one time, instead of 8.

### Added responsibilities of cytotechnologists

The key to the successful implementation of the imaging-system was the integration of cytotechnologists into the entire process, including extra FISH runs, quality control before and after FISH, scan setup, reclassification, and initial interpretation. The job description of the cytotechnologist specialist in our facility was changed to require that she/he must be able to perform quality rescreening and/or enumerate UroVysion FISH, and these individuals advanced by pay grade. The morphological expertise they offer has been critical to these activities, and could not be easily performed by someone without equivalent training. The relevant CLIA '88 sections that support the involvement of cytotechnologists in high complexity testing include 493.1481 (high complexity testing in cytology), 493.1483 (cytotechnologist qualifications), 493.1485 (cytotechnologist responsibilities), 493.1487 (testing personnel for high complexity testing), and 493.17 (test categorization).

### Expanded clinical and research applications

**Figure 8:**
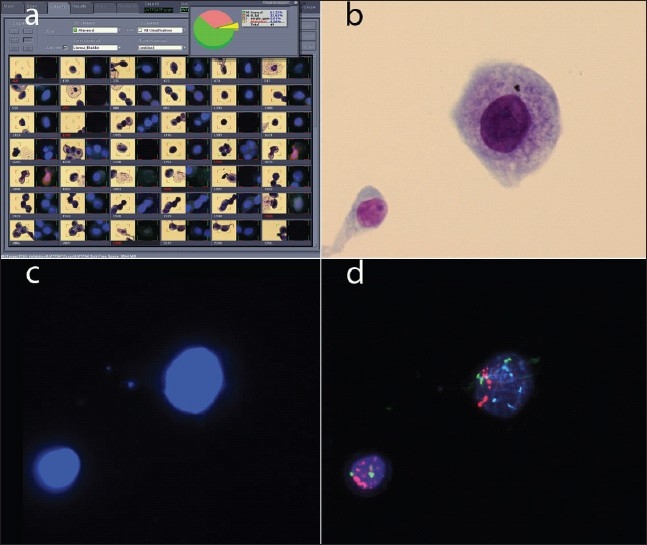
Target FISH. a) Gallery for a target FISH case, showing dual images of each target cell. The brightfield image is on the left; the DAPI image is on the right. b) Brightfield image of a cluster of four cells. c) DAPI image of the same four cells. d) Red and aqua probe signals for the same four cells.

In addition to UroVysion FISH for the detection of bladder cancer, the BioView imaging system offers fluorescence or brightfield applications in other areas, including diagnostics for other cancer types. In one application, Target FISH, stained or immunolabeled cells of interest or concern are first identified by brightfield microscopy, and are then evaluated by FISH by relocating the target cells in interactive or automated mode [Figure 8]. Target FISH using the Duet imaging system has been applied to the study of bladder cancer,[16,17] multiple myeloma,[18] and lymphocytic leukemia.[19,20]

The system provides a platform for additional tests. We have validated the use of UroVysion FISH probes and the Duet system in an ancillary test for exfoliative pancreatobiliary cytology specimens. Additional FISH tests are being evaluated for use as adjunct tests for cytology for interpretation of various cancers, including mesothelioma.

### Improvements to accuracy

The validation study confirmed that Duet-aided interpretation is at least equivalent to manual interpretation, with good concordance between the two methods. Equivalence between manual and image-aided interpretation was the standard applied by the FDA in granting clearance for the system.[2] In our validation study that compared manual and image-aided interpretations, two cases initially interpreted as negative in a manual scan and positive in a Duet-aided scan, were resolved as positive on review. For these two manual false-negative cases, an image-aided scan made it possible to identify the cases as positive. Because interpretations between manual and image-aided screening are equivalent, sensitivity and other performance measures are not expected to differ between the two.

## CONCLUSIONS

### Imaging the future

Although the changes required to integrate the system into the cytology lab seemed daunting, in retrospect the transitions occurred smoothly, and have resulted in a much more streamlined process, with more than 1500 cases now evaluated using Duet-aided interpretation since December of 2007. Other changes in cytopathology, such as telecytology and whole-slide scanning, are on the horizon,[21] and the skill sets of cytotechnologists should prepare them to benefit from the new technological tools.

## COMPETING INTEREST STATEMENT BY ALL AUTHORS

No competing interest to declare by any of the authors.

## AUTHORSHIP STATEMENT BY ALL AUTHORS

Each author acknowledges that this final version was read and approved. All authors of this article declare that we qualify for authorship as defined by ICMJE http://www.icmje.org/#author. Each author has participated sufficiently in the work and take public responsibility for appropriate portions of the content of this article.
